# Incidence and risk factors for norovirus‐related diarrhea in Japanese geriatric intermediate care facilities: A prospective cohort study

**DOI:** 10.1111/ggi.14539

**Published:** 2023-01-20

**Authors:** Asae Suita, Satoko Ohfuji, Wakaba Fukushima, Kazuya Ito, Tetsuo Kase, Kyoko Kondo, Motoki Ishibashi, Hiroko Kumashiro, Shuji Kawai, Akifumi Deguchi, Hiroyuki Nakata, Kazuko Iba, Tetsuya Kita, Kazuhiko Kinugawa, Kazutaka Hamada, Mikio Fujimoto, Yoshio Furukawa, Etsuji Sowa, Hideo Nakazawa, Yoshio Hirota

**Affiliations:** ^1^ Department of Public Health Osaka City University Graduate School of Medicine Osaka Japan; ^2^ Research Center for Infectious Disease Sciences Osaka City University Graduate School of Medicine Osaka Japan; ^3^ College of Healthcare Management Fukuoka Japan; ^4^ Clinical Epidemiology Research Center Medical Co. LTA (SOUSEIKAI) Fukuoka Japan; ^5^ Management Bureau Osaka City University Hospital Osaka Japan; ^6^ PS Clinic Souseikai Fukuoka Japan; ^7^ Tatsumanosato Long‐Term Care Health Facility for the Elderly Osaka Japan; ^8^ Kouseien Long‐Term Care Health Facility for the Elderly Osaka Japan; ^9^ Keai Long‐Term Care Health Facility for the Elderly Osaka Japan; ^10^ Tsukumo Long‐Term Care Health Facility for the Elderly Osaka Japan; ^11^ Yuai Long‐Term Care Health Facility for the Elderly Osaka Japan; ^12^ Midorigaoka Long‐Term Care Health Facility for the Elderly Osaka Japan; ^13^ Greenlife Long‐Term Care Health Facility for the Elderly Osaka Japan; ^14^ Tamagushi‐sumire‐en Long‐Term Care Health Facility for the Elderly Osaka Japan; ^15^ Sungarden Fuchu Long‐Term Care Health Facility for the Elderly Osaka Japan; ^16^ Ikuwakai Himawari Long‐Term Care Health Facility for the Elderly Osaka Japan; ^17^ Osaka City Long‐Term Care Health Facility for the Elderly Otoshiyori Sukoyaka Center Nanbukan Osaka Japan

**Keywords:** cohort study, diarrhea, intermediate care facility, Japan, norovirus

## Abstract

**Aim:**

The risk of developing infectious diarrhea among elderly residents at Japanese geriatric intermediate care facilities is unclear. We investigated the incidence rate and risk factors of norovirus‐related diarrhea at such facilities.

**Methods:**

This prospective cohort study followed 1727 residents from November 2018 to April 2020 at 10 geriatric intermediate care facilities in Osaka, Japan regarding the occurrence of diarrhea. Resident data were collected from their medical records using structured forms at two to three of the following three time points: at recruitment, if they developed diarrhea, and when they left the facility. Residents who developed diarrhea were tested using rapid diagnostic tests for norovirus. Cox proportional hazard model was employed to hazard ratios (HRs) with 95% confidence intervals (CIs) to estimate the risk factors for norovirus‐related diarrhea.

**Results:**

During the study period, 74 residents developed diarrhea, 13 of whom were norovirus positive. The incidence rate of norovirus‐related diarrhea was 10.11 per 1000 person‐years (95% CI: 4.61–15.61). In terms of risk factors, people with care‐needs level 3 were at a higher risk for developing norovirus‐related diarrhea (adjusted HR [aHR] = 7.35, 95% CI: 1.45–37.30). Residents with hypertension (aHR = 3.41, 95% CI: 1.05–11.04) or stroke (aHR = 8.84, 95% CI: 2.46–31.83), and those who walked with canes (aHR = 16.68, 95% CI: 1.35–206.52) also had a significantly higher risk for norovirus‐related diarrhea.

**Conclusions:**

Throughout the study period, the incidence of development of diarrhea was low. Care‐needs level 3, stroke, hypertension and use of a cane were identified as risk factors for norovirus‐related diarrhea in Japanese geriatric intermediate care facilities. **Geriatr Gerontol Int 2023; 23: 179–187**.

## Graphical abstract

The study aimed to investigate the incidence rate and risk factors of norovirus‐related diarrhea at Japanese geriatric intermediate care facilities. The incidence rate of norovirus‐related diarrhea was 10.11 per 1000 person‐years, and people with care‐needs level 3, stroke, hypertension and use of a cane were identified as risk factors for norovirus‐related diarrhea.

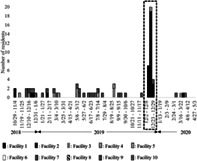

## Introduction

Japan has a super‐aging society, where the number of persons ≥65 years has been increasing, with this age group accounting for 29% of the population in 2020.^1^, ^2^ Accordingly, those who need residential care services have also been increasing. To address this situation, long‐term care insurance has been launched in Japan since 2000.^3^

Geriatric intermediate care facilities, which provide facility‐based care services, have special features, including residents receiving medical care from a full‐time doctor, in addition to nursing care and rehabilitation for their return to home. At these facilities, however, two to four residents usually live in the same room, which increases their risk of contracting infectious diseases and might lead to cluster outbreaks.^4^, ^5^, ^6^ Norovirus is the leading cause of outbreaks of acute viral gastroenteritis,^7^ with the attack rate of norovirus outbreaks of between 3% and 45% at long‐term care facilities.^8^ Moreover, facility residents often have many underlying diseases and poor physical functions,^9^ and once infected, they are likely to develop serious conditions, including dehydration, pulmonary aspiration of vomitus and even death.^10^, ^11^ A previous review showed a case fatality rate among the institutionalized elderly of 0.3%–1.6%.^8^ Another study reported that when hospitalized for norovirus gastroenteritis, the norovirus‐related mortality among patients living in long‐term care facilities was about three‐fold higher than in patients living at home.^12^ However, the incidence of norovirus‐related diarrhea among institutionalized elderly residents in Japan is limited, because national surveillance on infectious diarrheas is based on reports from pediatric sentinel sites, and because subjects who develop diarrhea are not always tested for pathogens.^12^, ^13^ Thus, it is hard to estimate the actual morbidity and mortality of norovirus‐related diarrhea among institutionalized elderly residents.

This study aimed to estimate the incidence and risk factors for norovirus‐related diarrhea among residents at Japanese geriatric intermediate care facilities. In this study, we actively performed rapid diagnostic tests for norovirus in all subjects with diarrhea, to diagnose norovirus‐related diarrhea accurately. In addition, we also tested diarrhea subjects for *Clostridioides difficile (C. difficile)*, since *C. difficile* is also a potential pathogen in these facility residents.

## Methods

### Study design and participants

This prospective cohort study was conducted at 10 geriatric intermediate care facilities that belong to the Osaka Association of Geriatric Health Service Facilities. All residents at the participating facilities during November 1, 2018 and April 30, 2020 were enrolled, except for those who received “short stay” residential services for less than 1 month, or who were ostomate. These facilities cater to residents aged ≥65 years, or those aged 40–64 years with specified diseases, including dementia and cerebrovascular disorders, under the long‐term care insurance law in Japan.^4^

### Data collection

At the time of enrollment (i.e., November 1, 2018 for already institutionalized residents, or the date of admission for newly admitted residents), we asked the staff to complete a structured form about the following characteristics of the study subjects based on their medical records: sex, age, underlying diseases (i.e. dementia, hypertension, stroke, heart disease, diabetes, malignancy, kidney disease, thyroid disease, chronic lung disease and liver disease), care‐needs level, activities of daily living (ADL) grade, level of cognitive dysfunction, number of roommates, history of hospitalization in the previous year, tube feeding, pad usage, independence in walking (“independent,” “using a cane,” “using a walking frame” and “impossible”), and prescription drugs (i.e., anticancer drugs, steroids, non‐steroidal anti‐inflammatory drugs (NSAIDs), proton pump inhibitors (PPIs), laxatives and probiotics).

Care‐needs level, ADL grade and level of cognitive dysfunction are variables that indicate the level of assistance that residents require. These variables are determined based on Japanese local government criteria using a national standardized procedure when patients seek long‐term care.^3^ Care‐needs level, which allows for admission to geriatric intermediate care facilities, consists of five levels.^3^ People with care‐needs level 1 are the least dependent, and people with care‐needs level 5 are bedridden, cannot communicate fully, and cannot eat by themselves. ADL grade was evaluated using the “Independence Criteria of the Daily Life of the Impaired Elderly.”^14^ These criteria have five ranks as follows: independent, almost independent (rank J), requires assistance when outside (rank A), wheelchair mobility (rank B), and bedridden (rank C).^14^, ^15^ Level of cognitive dysfunction was evaluated using the criteria for “Rating of dementia.”^15^ These criteria consist of six ranks: independent, presence of some cognitive disorder but almost independent (rank I), independent with supervision (rank II), requires some nursing care (rank III), requires nursing care all day (rank IV) and requires medical care (rank M).^5^, ^15^

### Follow‐up survey and outcome definitions

All residents were followed up through their medical records from enrollment until April 30, 2020, developing diarrhea, or leaving the facility, whichever came first.

Diarrhea was defined as two or more unformed or watery stools in 24 h. Residents who regularly took laxatives or suppositories were regarded as developing diarrhea if they had a greater frequency of defecation than usual. All residents with diarrhea received rapid diagnostic tests for norovirus (Immunocatch Norovirus Plus [Immunocatch]; Eiken Chemical, Tokyo, Japan) and *C. difficile* (GE‐test immunochromato‐CD GDH/TOX mini [GE‐test]; Nissui, Tokyo, Japan). The sensitivity and specificity of these kits are 100% and 93% (Immunocatch),^16^ and 98.1% and 98.2% (GE‐test),^17^ respectively, when compared with immunochromatography. Patients with positive results on these tests were regarded as having norovirus‐related diarrhea or *C. difficile*‐related diarrhea, respectively. In general, the guideline defines diarrhea as more than two unformed stools in 24 h.^18^ However, since many residents suffer from constipation, we were concerned to overlook the diarrhea occurrence when using the definition of this guideline. We therefore applied the broader criteria to grasp the diarrhea occurrence as much as possible and to employ the diagnostic tests of norovirus and *C. difficile* thoroughly for all diarrhea cases. Such active testing also enabled distinguishing between a laxative‐effective condition and diseased diarrhea in patients on laxatives.

If residents left the facility during the study period, the facility staff provided information on the date of discharge and the reason for leaving (return home, transfer to other type of geriatric care facility, hospitalization, or death) using a structured questionnaire.

### Statistical analysis

The incidence rate of diarrhea was calculated per 10 000 person‐days as the number of residents with diarrhea divided by the total follow‐up period of all participants. For residents with repeated admissions to and discharges from the facility during the study period, person‐days at risk were counted only for the period for which they were institutionalized. When presenting the results, the incidence rate using 10 000 person‐days was converted to 1000 person‐years. The incidence rate of norovirus‐related diarrhea was calculated by the same procedure as that for diarrhea.

The following characteristics were selected as possible predictive variables to assess their association with diarrhea: sex, age, care‐needs level, ADL grade, level of cognitive dysfunction, underlying diseases, prescription drugs, number of roommates, history of hospitalization in the previous year, tube feeding, pad usage and independence in walking. According to the Japanese long‐term care insurance system and previous research, age was divided into three categories: <65 years, 65–84 years and ≥85 years.^5^, ^9^

The Cox proportional hazard model was used to calculate hazard ratios (HRs) with 95% confidence intervals (CIs) to estimate the risk factors for diarrhea. Since care‐needs levels are determined based on the patient's ADL grade and level of cognitive dysfunction, a strong association is expected between them.^5^ Therefore, care‐needs level was considered as the first priority in the variable selection. The multivariate model included sex, age and care‐needs level as adjustment variables. In consideration of diarrhea outbreaks, an analysis focusing on the facility where residents developed norovirus‐positive diarrhea was also conducted. All analyses used SAS version 9.4 software (SAS Institute, Cary, NC, USA).

### Ethics statement

According to the Japanese Ethical Guidelines for Medical and Health Research Involving Human Subjects, since the study utilized only their pre‐existing data, subjects were notified about conduct of this study by displaying a poster, and patient consent for participation was obtained using an opt out method.^19^ If their stool samples were tested, the patients' verbal consent was obtained prior to the testing.

This protocol was approved by the Ethics Committee of Osaka City University Graduate School of Medicine (no. 4162; date of approval: October 25, 2018), and was performed in accordance with the Declaration of Helsinki.

## Results

Among 1734 residents initially included in the study, seven residents with missing data of predictive variables were excluded, and the data of 1727 residents were analyzed. Their baseline characteristics are shown in Table 1. One‐third of the residents were male, and the median age was 86 years. Dementia was the most common underlying disease (61%), followed by hypertension (51%) and stroke (33%). Residents with care‐needs level 4 constituted about 28% of all residents. More than half the residents had a history of hospitalization in the previous year, and tube feeding was required by 80 residents (5%). Twenty percent of the residents needed supportive items when walking, such as a cane or walking frame. Regarding prescription drugs, 951 residents (55%) took laxatives, and 563 residents (33%) took PPIs.

**Table 1:** Baseline characteristics of all the geriatric intermediate care facility residents

	Residents (*N* = 1727)
Characteristics	*n* (%)
Sex	
Male	536 (31)
Age (years)	
Median (range)	86 (43–107)
<65	36 (2)
65–84	705 (41)
≥85	986 (57)
Underlying diseases	
Dementia	1060 (61)
Hypertension	887 (51)
Stroke	562 (33)
Heart disease	511 (30)
Diabetes	358 (21)
Malignancy	254 (15)
Kidney disease	152 (9)
Thyroid disease	82 (5)
Chronic lung disease	76 (4)
Liver disease	66 (4)
Care‐needs level	
Level 1	194 (11)
Level 2	328 (19)
Level 3	415 (24)
Level 4	483 (28)
Level 5	307 (18)
Grade of ADL	
Independent, Rank J	39 (2)
Rank A	530 (31)
Rank B	964 (56)
Rank C	194 (11)
Level of cognitive dysfunction	
Independent, Rank I	238 (14)
Rank II	808 (47)
Rank III	590 (34)
Rank IV, Rank M	91 (5)
Number of roommates	
1	186 (11)
2	114 (7)
3	117 (7)
4	1310 (76)
History of hospitalization in the previous year	
Yes	1037 (60)
Tube feeding	
Yes	80 (5)
Pad usage (night only or always)	
Yes	1454 (84)
Independence in walking	
Independent	187 (11)
Using a cane	88 (5)
Using a walking frame	251 (15)
Impossible	1201 (70)
Prescription drugs	
Anticancer drugs	6 (0.4)
Steroids	67 (4)
NSAIDs	134 (8)
PPIs	563 (33)
Laxatives	951 (55)
Probiotics	162 (9)

Abbreviations: ADL, activities of daily living; NSAIDs, non‐steroidal anti‐inflammatory drugs; PPIs, proton pump inhibitors.

**Figure 1:**
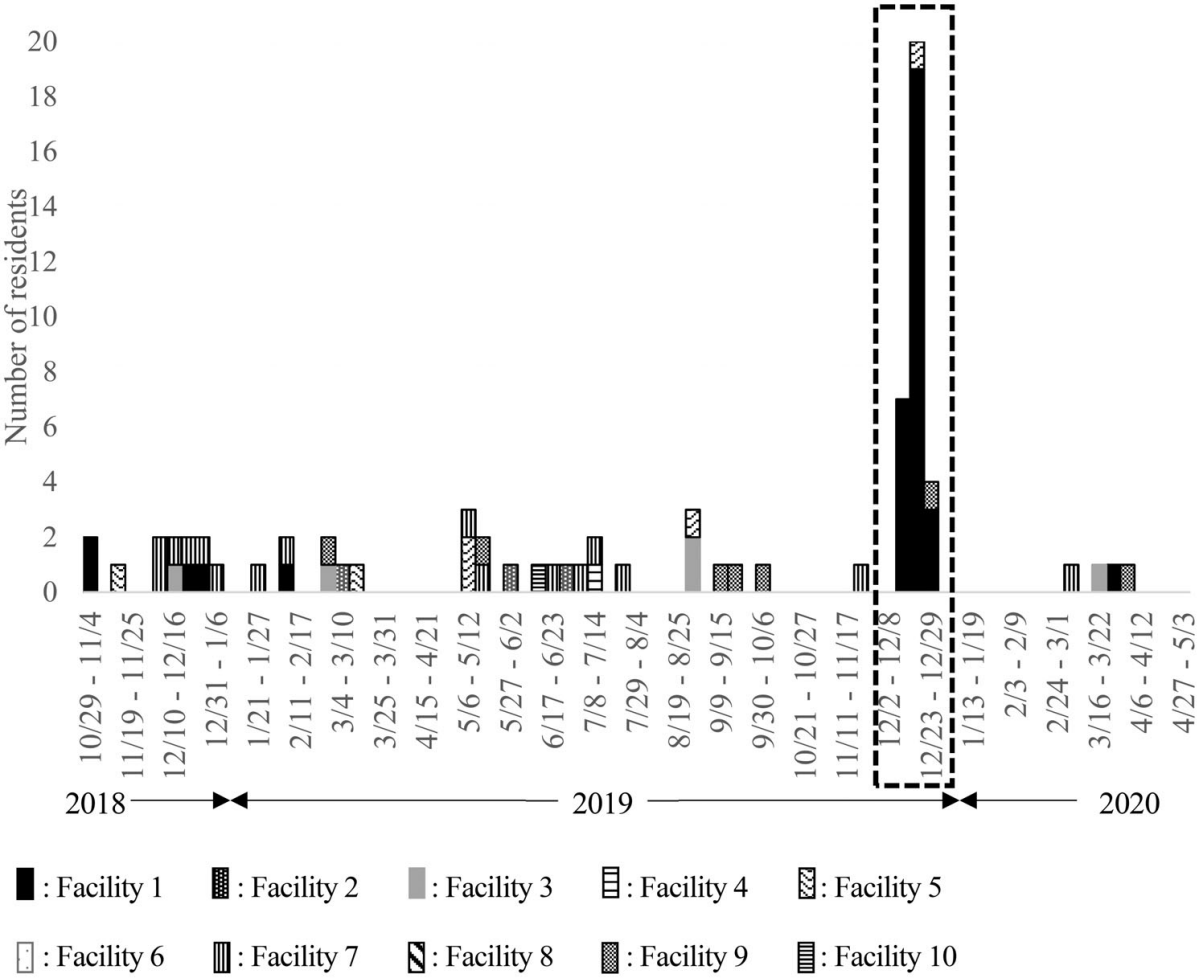
Distribution of residents who developed diarrhea by week and facility during the study period. During the study period (surrounded by the broken line), 13 residents were positive for norovirus. No residents were positive for norovirus at periods other than that indicated by the broken line.

Figure 1 indicates the distribution of residents who developed diarrhea by week. During the study period, 74 residents developed diarrhea (4.3%). The incidence rate of developing diarrhea was 59.03 per 1000 person‐years (95% CI: 45.58–72.48). Among these patients, 13 residents were positive for norovirus, all of which occurred only in December 2019 at a single facility. Other diarrhea cases were reported sporadically, and only one resident tested positive for *C. difficile*, although a retest was negative. Therefore, further analyses focused on norovirus‐related diarrhea as the study outcome. The incidence rate of developing norovirus‐related diarrhea was 10.11 (95% CI: 4.61–15.61) per 1000 person‐years.

Table 2 shows the relationship between background characteristics and the risk of developing norovirus‐related diarrhea. Sex and age did not have a significant relationship with the occurrence of norovirus‐related diarrhea. Compared with residents with care‐needs level 1 or 2, those with care‐needs level 3 had a significantly increased risk of developing norovirus‐related diarrhea (aHR = 7.35, 95% CI: 1.45–37.30).

**Table 2:** Relationship between risk of development of norovirus‐related diarrhea and selected background characteristics

Characteristics	*N*	Norovirus‐related diarrhea	Person‐years at risk	Incidence rate per 1000 person‐years					Facility where residents developed norovirus‐related diarrhea			
		*n* (%)			Crude HR (95% CI)		Adjusted HR^†^ (95% CI)		Crude HR (95% CI)		Adjusted HR^†^ (95% CI)	
Sex												
Male	536	2 (0.4)	344.9	5.80	1.00		1.00		1.00		1.00	
Female	1191	11 (0.9)	940.8	11.69	1.83	(0.40–8.27)	1.55	(0.33–7.16)	1.12	(0.25–5.10)	1.20	(0.26–5.56)
Age (years)												
<65	36	0 (0)	21.8	NA	NA		NA		NA		NA	
65–84	705	3 (0.4)	504.4	5.95	1.00		1.00		1.00		1.00	
≥85	986	10 (1.0)	759.6	13.17	2.12	(0.58–7.70)	1.85	(0.50–6.87)	1.91	(0.53–6.96)	1.05	(0.27–4.13)
Care‐needs level												
Level 1	194	0 (0)	133.0	NA	1.00		1.00		1.00		1.00	
Level 2	328	2 (0.6)	250.6	7.98								
Level 3	415	7 (1.7)	308.0	22.72	4.30	(0.89–20.71)	4.49	(0.93–21.63)	7.26	(1.49–35.44)	7.35	(1.45–37.30)
Level 4	483	4 (0.8)	358.6	11.16	2.13	(0.39–11.60)	2.13	(0.39–11.64)	1.33	(0.24–7.29)	1.43	(0.26–7.89)
Level 5	307	0 (0)	235.6	NA	NA		NA		NA		NA	
Grade of ADL^‡^												
Independent, Rank J	39	0 (0)	23.2	NA	1.00		1.00		1.00		1.00	
Rank A	530	3 (0.6)	399.4	7.51								
Rank B	964	9 (0.9)	713.4	12.62	1.74	(0.47–6.42)	1.82	(0.49–6.77)	1.68	(0.45–6.21)	1.78	(0.47–6.77)
Rank C	194	1 (0.5)	149.8	6.68	0.87	(0.09–8.33)	1.69	(0.17–17.22)	0.83	(0.09–8.05)	1.27	(0.11–14.87)
Level of cognitive dysfunction^‡^												
Independent, Rank I	238	1 (0.4)	154.7	6.46	1.00		1.00		1.00		1.00	
Rank II	808	8 (1.0)	596.1	13.42	2.03	(0.25–16.25)	1.86	(0.23–14.93)	0.54	(0.07–4.48)	0.50	(0.06–4.39)
Rank III	590	4 (0.7)	459.2	8.71	1.26	(0.14–11.29)	1.09	(0.12–9.96)	0.44	(0.05–4.18)	0.43	(0.04–4.36)
Rank IV, Rank M	91	0 (0)	75.7	NA	NA		NA		NA		NA	

Abbreviations: ADL, activities of daily living; CI, confidence interval; HR, hazard ratio; NA, not applicable.

^†^ Model includes sex, age and care‐needs level.

^‡^ Due to the presence of competing variables, the model includes sex, age, grade of ADL and level of cognitive dysfunction.

The risk of norovirus‐related diarrhea according to underlying diseases and prescription drugs is summarized in Table 3. Residents with hypertension or stroke had a significantly greater risk of norovirus‐related diarrhea (aHR of hypertension = 3.41, 95% CI: 1.05–11.04, aHR of stroke = 8.84, 95% CI: 2.46–31.83). Underlying diseases, such as malignancy, kidney disease and liver disease did not show any relationship with norovirus‐related diarrhea. Regarding prescription drugs, nine residents with norovirus‐related diarrhea took laxatives (69%), although there was no relationship with norovirus‐related diarrhea.

**Table 3:** Relationship between risk of development of norovirus‐related diarrhea and underlying diseases, and each prescription drugs

Characteristics		*N*	Norovirus‐related diarrhea	Person‐years at risk	Incidence rate per 1000 person‐years					Facility where residents developed norovirus‐related diarrhea			
			*n* (%)			Crude HR (95% CI)		Adjusted HR^†^ (95% CI)		Crude HR (95% CI)		Adjusted HR^†^ (95% CI)	
Underlying diseases													
Dementia	No	667	5 (0.8)	463.5	10.79	1.00		1.00		1.00		1.00	
	Yes	1060	8 (0.8)	822.2	9.73	0.87	(0.29–2.67)	0.86	(0.28–2.63)	0.49	(0.16–1.55)	0.53	(0.16–1.72)
Hypertension	No	840	5 (0.6)	600.7	8.32	1.00		1.00		1.00		1.00	
	Yes	887	8 (0.9)	685.0	11.68	1.39	(0.46–4.26)	1.35	(0.44–4.13)	2.52	(0.82–7.74)	3.41	(1.05–11.04)
Stroke	No	1165	6 (0.5)	862.8	6.95	1.00		1.00		1.00		1.00	
	Yes	562	7 (1.3)	422.9	16.55	2.33	(0.78–6.94)	3.88	(1.25–12.04)	3.09	(1.04–9.19)	8.84	(2.46–31.83)
Heart disease	No	1216	8 (0.7)	904.4	8.85	1.00		1.00		1.00		1.00	
	Yes	511	5 (1.0)	381.3	13.11	1.49	(0.49–4.54)	1.46	(0.47–4.50)	1.65	(0.54–5.06)	1.35	(0.39–4.67)
Diabetes	No	1369	10 (0.7)	1042.7	9.59	1.00		1.00		1.00		1.00	
	Yes	358	3 (0.8)	243.1	12.34	1.35	(0.37–4.90)	1.53	(0.41–5.65)	1.75	(0.48–6.41)	3.75	(0.77–18.31)
Malignancy	No	1473	13 (0.9)	1112.4	11.69	1.00		1.00		1.00		1.00	
	Yes	254	0 (0)	173.4	NA	NA		NA		NA		NA	
Kidney disease	No	1575	13 (0.8)	1163.0	11.18	1.00		1.00		1.00		1.00	
	Yes	152	0 (0)	122.8	NA	NA		NA		NA		NA	
Thyroid disease	No	1645	11 (0.7)	1220.2	9.02	1.00		1.00		1.00		1.00	
	Yes	82	2 (2.4)	65.6	30.49	3.30	(0.73–14.87)	2.48	(0.54–11.34)	2.90	(0.64–13.11)	1.53	(0.32–7.29)
Chronic lung disease	No	1651	12 (0.7)	1237.4	9.70	1.00		1.00		1.00		1.00	
	Yes	76	1 (1.3)	48.3	20.70	2.13	(0.28–16.38)	2.34	(0.30–18.29)	4.30	(0.55–33.89)	4.38	(0.51–37.79)
Liver disease	No	1661	13 (0.8)	1227.2	10.59	1.00		1.00		1.00		1.00	
	Yes	66	0 (0)	58.5	NA	NA		NA		NA		NA	
Prescription drugs													
Anticancer drugs	No	1721	13 (0.8)	1283.5	10.13	1.00		1.00		1.00		1.00	
	Yes	6	0 (0)	2.3	NA	NA		NA		NA		NA	
Steroids	No	1660	13 (0.8)	1233.0	10.54	1.00		1.00		1.00		1.00	
	Yes	67	0 (0)	52.8	NA	NA		NA		NA		NA	
NSAIDs	No	1593	12 (0.8)	1184.3	10.13	1.00		1.00		1.00		1.00	
	Yes	134	1 (0.8)	101.5	9.85	0.95	(0.12–7.31)	0.91	(0.12–7.11)	1.71	(0.22–13.30)	1.24	(0.15–10.00)
PPIs	No	1164	12 (1.0)	909.5	13.19	1.00		1.00		1.00		1.00	
	Yes	563	1 (0.2)	376.3	2.66	0.21	(0.03–1.65)	0.22	(0.03–1.73)	1.36	(0.17–10.72)	2.74	(0.30–24.89)
Laxatives	No	776	4 (0.5)	542.8	7.37	1.00		1.00		1.00		1.00	
	Yes	951	9 (1.0)	742.9	12.11	1.56	(0.48–5.06)	1.65	(0.51–5.38)	1.37	(0.42–4.46)	1.07	(0.31–3.67)
Probiotics	No	1565	12 (0.8)	1178.2	10.19	1.00		1.00		1.00		1.00	
	Yes	162	1 (0.6)	107.6	9.30	0.94	(0.12–7.19)	1.11	(0.14–8.57)	2.18	(0.28–16.81)	4.30	(0.40–46.78)

Abbreviations: CI, confidence interval; HR, hazard ratio; NA, not applicable; NSAIDs, non‐steroidal anti‐inflammatory drugs; PPIs, proton pump inhibitors.

^†^ Model includes sex, age and care‐needs level.

Table 4 shows the association between the risk of developing norovirus‐related diarrhea and selected variables related to daily life. After adjustment for sex, age and care‐needs level, residents who used pads had a significantly lower risk of developing norovirus‐related diarrhea (aHR: 0.14, 95% CI: 0.05–0.45), although the association was not significant in the analysis of only subjects at the facility where residents developed norovirus‐related diarrhea. In contrast, residents who walked using a cane had a more than 10‐fold risk for norovirus‐related diarrhea, with statistical significance (aHR: 16.68, 95% CI: 1.35–206.52).

**Table 4:** Relationship between risk of development of norovirus‐related diarrhea and each variable related to lifestyle behaviors

Characteristics	*N*	Norovirus‐related diarrhea	Person‐years at risk	Incidence rate per 1000 person‐years					Facility where residents developed norovirus‐related diarrhea			
		*n* (%)			Crude HR (95% CI)		Adjusted HR^†^ (95% CI)		Crude HR (95% CI)		Adjusted HR^†^ (95% CI)	
Number of roommates												
1	186	0 (0)	119.6	NA	NA		NA		NA		NA	
2	114	0 (0)	75.7	NA	NA		NA		NA		NA	
3	117	3 (2.6)	102.9	29.16	1.00		1.00		1.00		1.00	
4	1310	10 (0.8)	987.5	9.11	0.38	(0.10–1.38)	0.37	(0.10–1.36)	0.66	(0.18–2.42)	0.82	(0.21–3.17)
History of hospitalization in the previous year												
No	690	9 (1.3)	616.5	14.60	1.00		1.00		1.00		1.00	
Yes	1037	4 (0.4)	669.2	5.98	0.46	(0.14–1.49)	0.48	(0.15–1.58)	1.14	(0.34–3.85)	1.20	(0.34–4.23)
Tube feeding												
No	1647	12 (0.7)	1218.2	9.85	1.00		1.00		1.00		1.00	
Yes	80	1 (1.3)	67.5	14.81	1.36	(0.18–10.48)	6.82	(0.69–67.39)	1.01	(0.13–7.80)	6.49	(0.59–70.82)
Pad usage												
No	273	7 (2.6)	202.9	34.50	1.00		1.00		1.00		1.00	
Used (night only or always)	1454	6 (0.4)	1082.8	5.54	0.16	(0.06–0.49)	0.14	(0.05–0.45)	0.29	(0.10–0.86)	0.46	(0.12–1.78)
Independence in walking												
Independent	187	1 (0.5)	145.4	6.88	1.00		1.00		1.00		1.00	
Using a cane	88	2 (2.3)	62.6	31.97	5.45	(0.49–60.23)	5.80	(0.52–64.33)	11.80	(1.06–130.95)	16.68	(1.35–206.52)
Using a walking frame	251	0 (0)	191.7	NA	NA		NA		NA		NA	
Impossible	1201	10 (0.8)	886.1	11.29	1.69	(0.22–13.22)	1.58	(0.19–13.10)	2.87	(0.37–22.46)	5.49	(0.54–55.88)

Abbreviations: CI, confidence interval; HR, hazard ratio; NA, not applicable.

^†^ Model includes sex, age and care‐needs level.

## Discussion

The present cohort study at Japanese geriatric intermediate care facilities demonstrated that the incidence rate of norovirus‐related diarrhea is 10.11 per 1000 person‐years, and that four characteristics of residents are possible risk factors for norovirus‐related diarrhea: care‐needs level 3, stroke, hypertension and use of a cane for walking.

The incidence rate of norovirus‐related diarrhea in the present study subjects was much lower than that previously reported in a prospective cohort study at care homes in England, which found an incidence rate of 225.2 per 1000 person‐years.^20^ Although the incidences vary according to the study season, location and population, the difference between studies can partly be explained by the following three reasons. First, epidemics of infectious gastroenteritis in the community were low during the study seasons,^21^ which would have affected our results. At the end of the study period in 2020, in particular, due to the emergence of the global COVID‐19 pandemic, protective measures for infectious diseases at the facilities might have been more stringent. The second reason might have been derived from a notable feature of Japanese geriatric intermediate care facilities; since these facilities are managed by full‐time doctors and nurses, they might take more strict preventive measures against infectious diseases, including norovirus‐related diarrhea. In fact, a previous study indicated that Japanese geriatric intermediate care facilities pay more careful attention to dealing with healthcare facility‐related infections than Japanese intensive care homes.^22^ These characteristics may also explain why there was no relationship between the number of roommates and the occurrence of diarrhea. Third, we conducted tests for norovirus in all patients with diarrhea, which might have led to early detection of the index case in the facility and earlier disease control.

Residents with care‐needs level 3 were found to have a higher risk for norovirus‐related diarrhea. Typically, patients with care‐needs level 3 can walk with supportive items and can communicate with others.^4^ Compared with those with care‐needs level 4 or 5, they are likely to have a wider range of activities and come in contact with more people, leading to more opportunities for exposure to pathogens through contact, including norovirus.

The association between hypertension and norovirus‐related diarrhea might be explained by the immune condition of subjects with hypertension.^23^ Patients with hypertension have sympathetic nerve activation and higher levels of noradrenaline secretion, which suppresses the activation of T cells and cytokine production in their spleen,^24^ which might make patients more susceptible to developing infectious diseases.

In relation to the association between stroke and the risk of norovirus‐related diarrhea, a similar mechanism as with hypertensive patients might be involved, because the incidence of stroke is higher among patients with hypertension. In a previous study focusing on immune function in post‐stroke patients, two‐thirds of subjects had a history of hypertension.^25^ In the present study as well, 56% of residents with stroke had hypertension. Besides, dysfunction of neutrophils and immunodepression mediated by deactivation of monocytes might predispose post‐stroke patients to infections.^25^, ^26^ Another possible reason for the causality of these associations could be that some patients with hypertension or stroke receive assistance in their ADLs, such as meal assistance. Although these associations were observed after controlling for care‐needs level, residual confounding due to each type of daily life assistance might have remained. Besides, as far as we know, the association between hypertension or stroke and infectious diarrhea is very limited.^27^, ^28^ This suggests that it would be prudent to have reservations about these positive relationships.

In terms of cane usage, a retrospective cohort study in Hong Kong nursing homes indicated that residents who are dependent on a wheelchair for mobility have an increased risk of developing norovirus gastroenteritis.^29^ The authors suggested that indirectly touching surfaces contaminated with norovirus via the wheel of the wheelchair accounted for the higher risk, representing a fomite transmission pathway.^30^ In the present study facilities, since the canes belonged to each resident and were not shared, the association between norovirus‐related diarrhea and cane usage appeared to reflect indirect contact via walls touched by residents with infectious diarrhea.

Our study is noteworthy for the following two reasons. First, this study was conducted over a period of 1.5 years, which included two epidemic seasons for infectious gastroenteritis. We confirmed that the incidence of norovirus‐related diarrhea in the study facilities was in parallel with epidemics in the community in both seasons. Second, in this prospective and active follow‐up survey, all residents who developed diarrhea were tested for norovirus using kits with high sensitivity and specificity, so that the actual incidence rate of norovirus‐related diarrhea could be calculated.

However, our study has certain limitations, which would suggest caution when interpreting the results. First, although we adjusted for possible confounders, the presence of residual confounding could not be denied. Due to the low incidence of norovirus‐related diarrhea, multivariate models included an extremely limited number of factors as confounders, such as sex, age and care‐needs level. Second, since all residents who developed norovirus‐related diarrhea lived in the same facility, the risk factors presented in the study might also have been affected by the characteristics of the residents in this facility. To minimize this effect, however, we separately evaluated the results of residents at a facility where those with norovirus‐related diarrhea lived. Third, since this study was conducted at a single prefecture in Japan, the results cannot be generalized. Therefore, future studies in other regions or other seasons are required to confirm the validity of the study results.

In conclusion, the present study provides evidence related to norovirus‐related diarrhea in older people who live at geriatric intermediate care facilities, who are not reported on in national surveillance systems and have rarely been focused on in previous studies. The incidence rate of norovirus‐related diarrhea among residents at Japanese geriatric intermediate care facilities during the study period was lower than expected. However, people with care‐needs level 3, hypertension, stroke and those who used a cane for walking had a significantly higher risk for developing norovirus‐related diarrhea. These results highlight the disease burden of norovirus‐related diarrhea in geriatric intermediate care facilities. To prevent a norovirus outbreak in such facilities, the staff should continue taking standard precautions on a daily basis, including hygiene control, particularly during nursing care of residents with high risk factors, and thoroughly disinfecting the handrails and door handles touched by residents using canes with alcohol. In addition to these basic precautions, when a resident develops suspected norovirus‐positive diarrhea or vomit, following the facilities' precautions, the handrails and door handles should also be disinfected with sodium hypochlorite as soon as possible, since disinfection with alcohol is known to be less effective for norovirus.

## Disclosure statement

The authors declare no conflict of interest.

## Author contributions

AS contributed to study design, statistical analysis, data interpretation and manuscript writing (Author Contribution Index [ACI]: 1.77). SO contributed to study concept, study design, data management and data interpretation (ACI: 1.54). WF, KI_1_, TK_1_ and KK_1_ contributed to study concept, study design and data interpretation (ACI: 1.21). MI, HK, SK and YH contributed to study concept and study design (ACI: 1). AD and HN_1_ contributed to study concept, study design and data collection (ACI: 1). KI_2_, TK_2_, KK_2_, KH, MF, YF, ES and HN_2_ contributed to data acquisition (ACI:0.79). All authors provided comments on the drafts and have read and approved the final manuscript.

## Data Availability

The data that support the findings of this study are available on request from the corresponding author. The data are not publicly available due to ethical restrictions.

## References

[1] Muramatsu N , Akiyama H . Japan: Super‐aging society preparing for the future. Gerontologist 2011; 51: 425–432. 10.1093/geront/gnr067. 21804114

[2] Statistics Bureau of the Ministry of Internal Affairs and Communications, Japan . Population Estimates, Japan. 2020. [Cited February 5, 2021]. Available from the URL: https://www.stat.go.jp/data/jinsui/pdf/202010.pdf. (in Japanese).

[3] Campbell JC , Ikegami N . Long‐term care insurance comes to Japan. Health Affairs 2000; 19: 26–39. 10.1377/hlthaff.19.3.26. 10812779

[4] Morita K , Ono S , Ishimaru M , Matsui H , Naruse T , Yasunaga H . Factors affecting discharge to home of geriatric intermediate care facility residents in Japan. J Am Geriatr Soc 2018; 66: 728–734. 10.1111/jgs.15295. 29461630

[5] Nakanishi M , Hattori K , Nakashima T , Sawamura K . Health care and personal care needs among residents in nursing homes, group homes, and congregate housing in Japan: why does transition occur, and where can the frail elderly establish a permanent residence? J Am Med Dir Assoc 2014; 15: e1–e6. 10.1016/j.jamda.2013.07.006. 23981788

[6] Hamada S , Ohno Y , Kojima T , Ishii S , Okochi J , Akishita M . Prevalence of cytochrome P450‐mediated potential drug‐drug interactions in residents of intermediate care facilities for older adults in Japan. Geriatr Gerontol Int 2019; 19: 513–517. 10.1111/ggi.13652. 30912281

[7] Utsumi M , Makimoto K , Quroshi N , Ashida N . Types of infectious outbreaks and their impact in elderly care facilities: a review of the literature. Age Ageing 2010; 39: 299–305. 10.1093/ageing/afq029. 20332371

[8] Lindsay L , Wolter J , De Coster I , Van Damme P , Verstraeten T . A decade of norovirus disease risk among older adults in upper‐middle and high income countries: a systematic review. BMC Infect Dis 2015; 15: 425. 10.1186/s12879-015-1168-5. 26467099 PMC4606836

[9] Šubelj M , Učakar V . An outbreak of acute gastroenteritis associated with group a rotavirus in long‐term care facility in Slovenia. Wien Klin Wochenschr 2015; 127: 415–420. 10.1007/s00508-014-0672-8. 25447968

[10] Rao K , Micic D , Chenoweth E *et al*. Poor functional status as a risk factor for severe Clostridium difficile infection in hospitalized older adults. J Am Geriatr Soc 2013; 61: 1738–1742. 10.1111/jgs.12442. 24083842 PMC3801297

[11] Mattner F , Sohr D , Heim A , Gastmeier P , Vennema H , Koopmans M . Risk groups for clinical complications of norovirus infections: an outbreak investigation. Clin Microbiol Infect 2006; 12: 69–74. 10.1111/j.1469-0691.2005.01299.x. 16460549

[12] Ohfuji S , Kondo K , Ito K *et al*. Nationwide epidemiologic study of norovirus‐related hospitalization among Japanese older adults. BMC Infect Dis 2019; 19: 400. 10.1186/s12879-019-4007-2. 31072305 PMC6506929

[13] Kato H , Senoh M , Honda H *et al*. Clostridioides (Clostridium) difficile infection burden in Japan: a multicenter prospective study. Anaerobe 2019; 60: 102011. 10.1016/j.anaerobe.2019.03.007. 30872073

[14] Tokuhashi Y , Ajiro Y , Umezawa N . Outcomes of posterior fusion using pedicle screw fixation in patients >or=70 years with lumbar spinal canal stenosis. Orthopedics 2008; 31: 1096. 19226091

[15] Ministry of Health, Labour and Welfare . Long‐term care insurance in Japan, Survey of institutions and establishments for long‐term care, 2010; Explanation of Main Terms. 2012. [Cited February 5, 2021]. Available from the URL: https://www.mhlw.go.jp/english/database/db-hss/dl/siel-2010-04.pdf. (in Japanese).

[16] Package insert of IMMUNOCATCH® Norovirus Plus . 2nd ed. (in 2018). [Cited April 13, 2021]. Available from the URL: https://www.eiken.co.jp/POCT/products/noro_plus/pdf/noro_Plus_tenpu2.pdf. (in Japanese).

[17] Package insert of GE‐test immunochromato‐CD GDH/TOX mini . 4th ed. (in 2018) and 5th ed. (in 2019). [Cited August 23, 2022] Available from the URL: https://www.info.pmda.go.jp/downfiles/ivd/PDF/530121_22800EZX00028000_A_01_08.pdf. (in Japanese).

[18] McDonald LC , Gerding DN , Johnson S *et al*. Clinical practice guidelines for Clostridium difficile infection in adults and children: 2017 update by the Infectious Diseases Society of America (IDSA) and Society for Healthcare Epidemiology of America (SHEA). Clin Infect Dis 2018; 66: e1–e48. 10.1093/cid/cix1085. 29462280 PMC6018983

[19] Ministry of Health, Labour and Welfare . Ethical Guidelines for Medical and Health Research Involving Human Subjects. [Cited January 4, 2022]. Available from the URL: https://www.mhlw.go.jp/file/06-Seisakujouhou-10600000-Daijinkanboukouseikagakuka/0000080278.pdf.

[20] Inns T , Pulawska‐Czub A , Harris JP *et al*. Prospective cohort study to investigate the burden and transmission of acute gastroenteritis in care homes: epidemiological results. BMJ Open 2019; 9: e033239. 10.1136/bmjopen-2019-033239. PMC6924874 31818842

[21] Osaka Institute of Public Health . The information of infectious gastroenteritis, Comparison the number of infectious gastroenteritis cases reported per sentinels in Osaka Prefecture during past 10 years; 2020. [Cited February 5, 2021]. Available from URL: http://www.iph.pref.osaka.jp/infection/noro/noro.html. (in Japanese).

[22] Takushima H , Yamamoto K , Tokuzumi K , Moritsuka M . Infection control in long‐term care facilities of present situation‐results from an investigation of managers at long‐term care facilities. J Health Sci 2013; 10: 25–34 (in Japanese).

[23] Case AJ , Zimmerman MC . Sympathetic‐mediated activation versus suppression of the immune system: consequences for hypertension. J Physiol 2016; 594: 527–536. 10.1113/jp271516. 26830047 PMC4930069

[24] Marvar PJ , Thabet SR , Guzik TJ *et al*. Central and peripheral mechanisms of T‐lymphocyte activation and vascular inflammation produced by angiotensin II‐induced hypertension. Circ Res 2010; 107: 263–270. 10.1161/circresaha.110.217299. 20558826 PMC2921936

[25] Haeusler KG , Schmidt WU , Föhring F *et al*. Cellular immunodepression preceding infectious complications after acute ischemic stroke in humans. Cerebrovasc Dis 2008; 25: 50–58. 10.1159/000111499. 18033958

[26] van Gemmeren T , Schuppner R , Grosse GM *et al*. Early post‐stroke infections are associated with an impaired function of neutrophil granulocytes. J Clin Med 2020; 9: 872. 10.3390/jcm9030872. 32209993 PMC7141520

[27] Trifan A , Girleanu I , Stanciu C *et al*. Clostridium difficile infection in hospitalized octogenarian patients. Geriatr Gerontol Int 2018; 18: 315–320. 10.1111/ggi.13186. 29139189

[28] Cadena J , Thompson GR 3rd , Patterson JE *et al*. Clinical predictors and risk factors for relapsing Clostridium difficile infection. Am J Med Sci 2010; 339: 350–355. 10.1097/MAJ.0b013e3181d3cdaa. 20224312

[29] Lin H , Ng S , Chan S *et al*. Institutional risk factors for norovirus outbreaks in Hong Kong elderly homes: a retrospective cohort study. BMC Public Health 2011; 11: 297. 10.1186/1471-2458-11-297. 21569308 PMC3103461

[30] Xiao S , Tang JW , Li Y . Airborne or fomite transmission for norovirus? A Case study revisited. Int J Environ Res Public Health 2017; 14: 1571. 10.3390/ijerph14121571. 29240665 PMC5750989

